# Monitoring corn stover processing by the fungus *Ustilago maydis*

**DOI:** 10.1186/s40643-024-00802-3

**Published:** 2024-09-14

**Authors:** Stefan Robertz, Magnus Philipp, Kerstin Schipper, Paul Richter, Katharina Miebach, Jorgen Magnus, Markus Pauly, Vicente Ramírez

**Affiliations:** 1 Institute for Plant Cell Biology and Biotechnology, Cluster of Excellence on Plant Sciences, Heinrich Heine University Düsseldorf, 40225 Düsseldorf, Germany; 2 Institute for Microbiology, Heinrich Heine University Düsseldorf, 40225 Düsseldorf, Germany; 3 Aachener Verfahrenstechnik – Chair of Biochemical Engineering, RWTH Aachen University, 52074 Aachen, Germany; 4 Bioeconomy Science Center (BioSC), Forschungszentrum Jülich, 53435 Jülich, Germany; 5 Present Address: Institute for Biotechnology and Foodscience, Norwegian University of Science and Technology, 7034 Gløshaugen, Trondheim, Norway

**Keywords:** Lignocellulose utilization, Corn stover, *Ustilago maydis*, Bioconversion, Online monitoring

## Abstract

A key aspect of sustainable bioeconomy is the recirculation of renewable, agricultural waste streams as substrates for microbial production of high-value compounds. One approach is the bioconversion of corn stover, an abundant maize crop byproduct, using the fungal maize pathogen *Ustilago maydis. U. maydis* is already used as a unicellular biocatalyst in the production of several industrially-relevant compounds using plant biomass hydrolysates. In this study, we demonstrate that *U. maydis* can grow using untreated corn stover as its sole carbon source. We developed a small-scale bioreactor platform to investigate *U. maydis* processing of corn stover, combining online monitoring of fungal growth and metabolic activity profiles with biochemical analyses of the pre- and post-fermentation residues. Our results reveal that *U. maydis* primarily utilizes soluble sugars *i.e.,* glucose, sucrose and fructose present in corn stover, with only limited exploitation of the abundant lignocellulosic carbohydrates. Thus, we further explored the biotechnological potential of enhancing *U. maydis´* lignocellulosic utilization. Additive performance improvements of up to 120 % were achieved when using a maize mutant with increased biomass digestibility, co-fermentation with a commercial cellulolytic enzyme cocktail, and exploiting engineered fungal strains expressing diverse lignocellulose-degrading enzymes. This work represents a key step towards scaling up the production of sustainable compounds from corn stover using *U. maydis* and provides a tool for the detailed monitoring of the fungal processing of plant biomass substrates.

**Graphical abstract:**

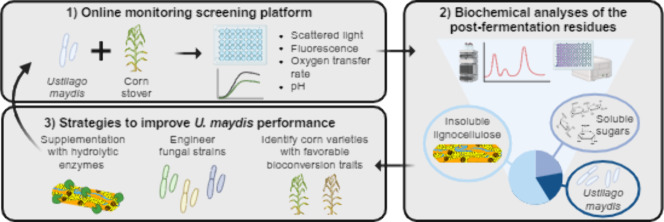

**Supplementary Information:**

The online version contains supplementary material available at 10.1186/s40643-024-00802-3.

## Background

Bioconversion of agricultural residues such as corn stover holds significant potential as a sustainable production process of environmentally friendly chemicals with high added value (Pauly and Keegstra 2008). One interesting approach is the use of lignocellulose-degrading fungi as biocatalysts. These microbes are naturally equipped with enzymatic systems capable of hydrolyzing the complex carbohydrates present in lignocellulosic feedstocks into fermentable carbohydrates, which can then be converted into desired products. The dimorphic fungus *Ustilago maydis*, a natural maize pathogen, is an excellent candidate for the direct bioconversion of corn stover, combining a sturdy lignocellulolytic potential (Cano-Canchola et al. 2000; Couturier et al. 2012; Reyre et al. 2022) with excellent adaptability to fermentation processes on an industrial scale (Feldbrügge et al. 2013; Beck and Zibek 2020). *U. maydis* naturally produces a wide range of biotechnologically relevant metabolites, including organic acids, polyols, lipids, and biosurfactants (Wierckx et al. 2021). A well-annotated genome and robust genome editing toolkit have enabled the development of several strains with substantial yield enhancements (Paulino et al. 2017). Under laboratory conditions, *U. maydis* can be propagated in a yeast-like, haploid form compatible with a precise monitoring of growth performance in submerged cultivations (Zambanini et al. 2017; Hartmann et al. 2018). Additionally, the fungus can utilize a variety of carbon sources, and demonstrates an exceptional tolerance to media impurities and hydromechanical stress, desirable attributes for a successful fermentation scale-up (Feldbrügge et al. 2013; Wierckx et al. 2021; Volkmar et al. 2023).

The main obstacles for the direct bioconversion of corn stover using *U. maydis* are the structural complexity and compositional heterogenicity of the material. Corn stover lignocellulose is mainly composed of cellulose (~ 35 % w/w), glucuronoarabinoxylan (GAX) (~ 20 % w/w), and lignin (~ 12 % w/w) (Vogel 2008). Cellulose is an unsubstituted homopolysaccharide consisting of β-1,4-linked D-glucopyranosyl units. A large portion of cellulose chains aggregate to form crystalline, highly organized microfibrils through extensive inter- and intra-molecular hydrogen bonds, which impedes cellulose degradation. Only amorphous cellulose sections, *i.e.* regions where the ordered organization is lost, are easily accessible to hydrolytic enzymes (Perrot et al. 2022). GAX, the primary matrix polysaccharide in grasses, is characterized by a highly *O*-acetylated linear β-1,4-linked D-xylopyranose backbone which can be further decorated with pentose, hexose, and (methyl)uronic or hydroxycinnamic acid sidechains (Rennie and Scheller 2014). This structural heterogeneity hinders enzymatic degradation, and multiple enzymes with specific substrate recognition and cleavage capabilities are needed to efficiently convert GAX (Verbruggen et al. 1998; Rojas-Pérez et al. 2022). Lignin is an aromatic heteropolymer consisting mainly of guaiacyl, syringyl and/or p-hydroxyphenyl monolignol subunits. Among all the lignocellulosic components, the enzymatic depolymerization of lignin is the most challenging, due to its highly hydrophobic and heterogeneous nature (Hatfield et al. 2017; Janusz et al. 2017; Gao et al. 2020). Together, these structural and compositional complexity factors add to the recalcitrance of corn stover lignocellulose to enzymatic degradation, making a coordinated activity of multiple hydrolytic enzymes necessary.

As a widespread maize pathogen, the *U. maydis* genome encodes a wide set of enzymes with potential to degrade maize cell walls, which are required to penetrate plant tissues during its infection cycle (Kämper et al. 2006; Brefort et al. 2009). Over 80 *U. maydis* proteins potentially involved in lignocellulose degradation have been identified, including 33 secreted carbohydrate-active enzymes (CAZymes; (Drula et al. 2022)). While only a handful of these *U. maydis* CAZymes have been biochemically characterized, their predicted activities align with the degradation of key lignocellulosic elements present in corn stover such as cellulose and GAX (Mueller et al. 2008; Doehlemann et al. 2008; Couturier et al. 2012; Reyre et al. 2022). Indeed, the presence of corn tissue in the culture media stimulates *U. maydis* to produce enzymes with high lytic activities tested against individual cell wall polysaccharides (Cano-Canchola et al. 2000; Couturier et al. 2012). These findings support the notion of *U. maydis* as a fungus with significant biotechnological potential for plant biomass bioconversion (Feldbrügge et al. 2013; Geiser et al. 2016; Müller et al. 2018; Regestein et al. 2018; Schlembach et al. 2020). However, critical details, such as the specific carbohydrate nutrient sources utilized by the fungus and the efficiency of biomass degradation have yet to be determined.

In this study, we adapted a small-scale microtiter plate cultivation platform to monitor the performance of *U. maydis* as a unicellular biocatalyst for the degradation of corn stover. The method is based on the BioLector® and micro respiratory activity monitoring system (µRAMOS) technologies, allowing the growth of multiple cultures under constant conditions, while monitoring fungal growth and metabolism online. By combining the online monitoring with the analysis of the chemical composition of the post-metabolized lignocellulosic residue we achieved a comprehensive understanding not only of *U. maydis* growth and metabolic profiles growing on a complex lignocellulosic substrate, but also of the specific corn stover-derived carbohydrate substrates utilized by the fungus for its proliferation. Additionally, the applicability of this methodology for potential future process optimization is assessed.

## Methods

### Plant materials and growth

The maize lines B73 and *bm3* were grown in the greenhouse under a 16 h/8 h light/dark-regiment and temperatures between 20 °C and 25.6 °C. The plants were watered twice per day and fertilized twice per week with 0.5 % (v/v) Wuxal®. Senescent stems from 5-month-old plants were harvested, cut into 15 cm pieces and air-dried at 50 °C for 5 days.

## Biomass preparation

Individual dried stems from four B73 and six *bm3* plants were collected and processed as described in (Wang et al. 2023). In short, the material was ground into powder in a GM200 mixer mill (Retsch) and freeze-dried (Coolsafe system (Scanvac)). The powder was then milled in 2 mL screw cap tubes containing two 5 mm steel balls for 3 × 2 min at 30 Hz in a MM400 mixer mill (Retsch). The fine powder was resuspended in water (50 mg/mL) and sterilized by autoclaving (15 min, 121 °C). The particle size of the fine powder (42 ± 26 µm; Additional file 2: Figure S1) was determined by analyzing representative pictures taken with a Leica DM2000 microscope equipped with a Leica MC170HD camera and measuring the size of individual particles with ImageJ (Version 1.54).

## Fungal strains and corn stover fermentation

Unless otherwise stated, all fermentation experiments were performed with the *Ustilago maydis* strain MB215^Gfp^P_oma_bgl1. Therefore, the MB215P_oma_bgl1 strain described in (Geiser et al. 2016) was further modified to express a cytoplasmic green fluorescent protein (Gfp). This was achieved by stable genomic integration of the *gfp* open reading frame under control of the constitutive promoter P_*otef*_ into the *ip* locus using the integrative plasmid pOTEF-SG (Spellig et al. 1996) according to previously described protocols (Stock et al. 2012). MB215 was similarly engineered to express Gfp to generate MB215^Gfp^ and used as a control. The other two MB215 derivatives P_oma_egl1 and P_oma_xyn11A used were obtained from (Geiser et al. 2016).

For the *U. maydis* inoculum, overnight pre-cultures were inoculated with *U. maydis* grown on complete medium (CM; components listed in Additional file 1: Table S1) agar plates supplemented with 1 % (w/v) glucose (Glc) (Holliday 1974) and used to inoculate main cultures to an optical density (OD_600_) of 0.2. The main cultures were grown for 5 h, washed once, and resuspended in sterile water to a final OD_600_ of 1.5. MTP-R48-BOH 1 round well microtiter plates were filled with 1.5 mL suspension per well consisting of 600 µL corn stover (50 mg/mL), 750 µL 2 × CM without Glc, 50 µL sterile water and 100 µL *U. maydis* inoculum (final OD_600_ of 0.1). The plates were incubated at 28 °C with 1000 rpm constant shaking in a BioLector® device (Beckman Coulter Life Sciences, Aachen, Germany). To test the effect of Celluclast® addition 0.5 µL per mg plant biomass was included in the suspension. Scattered light was measured at a wavelength of 620 nm and a gain of 15. Additionally, Gfp fluorescence (λ_Ex._ = 488 nm; λ_Em._ = 520 nm, gain = 80), and pH (λ_Ex._ = 470 nm; λ_Em._ = 525 nm, gain = 23) were measured, with readings every 30 min. Scattered light and Gfp fluorescence values were normalized to the values obtained 2.5 h after inoculation, once the corn stover particles in the wells have reached homogeneous distribution. The respiration activity of the culture was measured in each well of the 48-well microtiter plate in form of the oxygen transfer rate (OTR) with the µRAMOS device as previously described (Flitsch et al. 2016; Ladner et al. 2016).

## Microscopy

For microscopy, samples of cultures growing on 1 × CM medium supplemented with 1 % (w/v) Glc or with 50 mg/ml corn stover in the BioLector® device at 28°C were analyzed after 16 h of incubation. Cells were immobilized on agarose patches (2 % final concentration) and visualized with a wide-field microscope setup from Visitron Systems, Axio Imager M1 (Visitron Systems GmbH, Puchheim, Germany) equipped with a Spot Pursuit CCD camera (Diagnostic Instruments, Sterling Heights, MI) and a 40 × objective lens (Plan Neofluar; NA 1.3, Carl Zeiss, Jena, Germany). Gfp signals were acquired using a HXP metal halide lamp (LEj, Jena, Germany) in combination with a filter set for Gfp (ET470/40BP, ET495LP, ET525/50BP). The microscopic system was operated with the software MetaMorph (Molecular Devices, version 7, Sunnyvale, USA).

## Residue analyses

The residual material after fungal fermentation was transferred to 2 mL screw-cap tubes, centrifuged, and the solid was separated from the liquor for compositional analyses. The liquor fraction was filtered through a PTFE membrane filter (0.2 µm) and used directly for soluble sugar analysis on a high-performance anion-exchange chromatography (HPAEC) (Metrohm or Knauer Azura) equipped with a CarboPac PA20 column (3 × 150 mm) and a PAD detector (Metrohm or Antec Scientific Decade Elite). The following gradient was used: 21 min 1 mM NaOH, 9 min 700 mM NaOH and 13 min 1 mM NaOH, with a flow rate of 0.5 mL/min. Fucose was used as an internal standard.

The solid residue was dried and alcohol-insoluble residue (AIR) was prepared as described in (Foster et al. 2010a). The simultaneous quantification of hemicellulosic monosaccharides and cellulose content in the solid, was performed according to (Wang et al. 2023). Fucose was used as internal standard, as this monosaccharide is absent in *U. maydis*-derived cell walls. After the acid hydrolysis, monosaccharide mixtures were separated using the same HPAEC system described above for the soluble sugars but using the following gradient: 23 min 2 mM NaOH, 7 min 490 mM NaOH, 3 min 700 mM NaOH, 24 min 2 mM NaOH, with a flow rate of 0.4 mL/min. Cell wall-bound acetate content was determined as described in (Ramírez et al. 2018). In short, 1 mg AIR material was incubated in 0.25 M NaOH at 25 °C for 1 h. After neutralization with HCl, acetic acid content was quantified using an Acetic Acid Assay Kit (Megazyme). The total amount of starch was quantified based on the Total Starch Kit (K-TSTA) method (Megazyme) with slight modifications. AIR (1 mg) was treated with a thermostable α-amylase for 15 min at 100 °C with inversion every 5 min. After cooling down, 50 µL amyloglucosidase were added, vortexed and incubated for 30 min at 50 °C. The glucose content was then quantified in the supernatant with the GOPOD method according to (Kraemer et al. 2021). Acetylbromide soluble lignin content was determined according to (Foster et al. 2010a). The shown data represent the averages and standard deviations of the fermentation experiments mentioned above. An overview of the individual measurements is shown in Additional file 1: Table S2.

For the estimation of *U. maydis* biomass material in the solid residue, first, *U. maydis* was grown in equivalent BioLector® conditions with 1 % (w/v) Glc as a carbon source instead of corn stover. Monosaccharide composition from the solid residue was determined as described for the plant-fungal mixtures. Under these conditions, galactose (Gal), Glc, mannose, glucosamine (GlcN), and ribose were detected (Additional file 2: Figure S2). The identity of the monosaccharides was confirmed by the alditol acetate method based on (Foster et al. 2010b). Alditol acetates were injected into a gas chromatograph (7890B, Agilent) equipped with a quadrupole electronic ionization mass analyzer (5977A). A Supelco SP-2380 (30 mm × 0.25 mm × 0.20 µm film thickness) column was used with a helium flow rate of 1.5 mL/min. The oven gradient followed this protocol: initial temperature 80 °C for 3 min followed by a ramp to 170 °C with 30 °C/min, afterwards a ramp to 240 °C with 4 °C/min, and a final hold at 240 °C for 15 min. In addition, a constant amount of corn stover AIR was mixed with increasing amounts of *U. maydis* AIR grown on Glc, and the GlcN content was quantified, establishing a linear correlation between *U. maydis* AIR and the detected GlcN content (Additional file 2: Figure S2). Finally, the GlcN content in the solid residue from fermented samples (mixture of plant and fungus) was used to estimate the amount of fungal material. Given that Gal and Glc are present in both plant and fungal material, conversion factors were applied to determine the proportion of these monosaccharides originating from each source based on the GlcN-estimated fungal material (Additional file 2: Figure S2).

## Mass balance in glass flasks

For quantitative determinations of the residual material, corn stover (variety B73) fermentations were also carried out in pre-weighed glass shake flasks (100 mm x 13 mm), with the same compositional setup used for the BioLector fermentations. The cultures were shaken horizontally at 300 rpm, 28 °C, angled at 44°. At the end of the fermentation, the flasks were centrifuged and the liquor was separated from the solid, which was dried by speedvac (Concentrator Plus, Eppendorf) for 60 min at 45 °C and quantified gravimetrically. Subsequently, AIR was prepared and the remaining solid was quantified gravimetrically. Solid analyses were conducted as described above.

## Results and Discussion

### *Ustilago* maydis can utilize corn stover

**Fig. 1:**
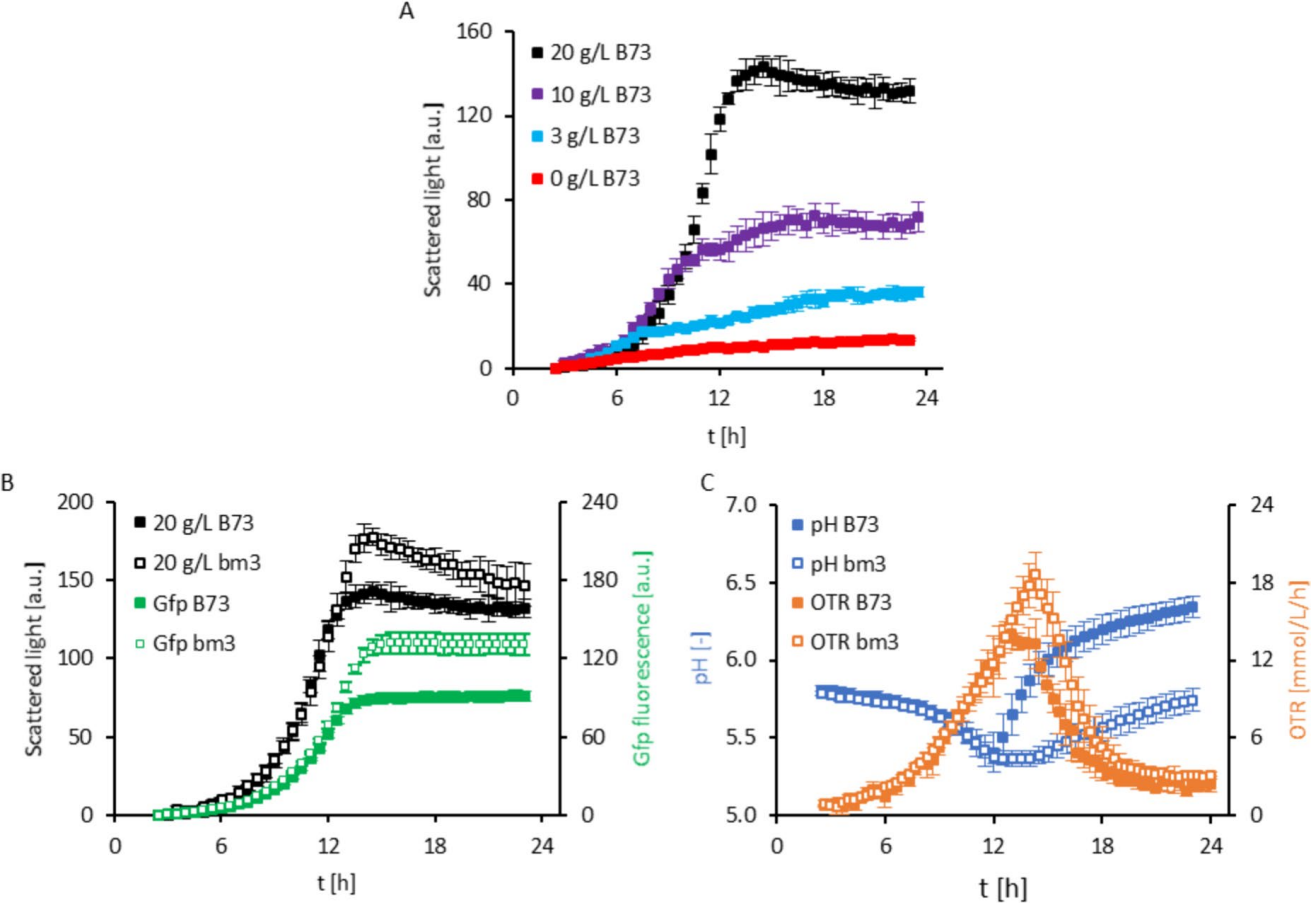
Online performance monitoring of *Ustilago maydis* on corn stover. (**A**) Scattered light reading of fungal growth in medium supplemented with 3 g/L (light blue), 10 g/L (purple) or 20 g/L (black) ground B73 corn stem material from 4 individual plants in comparison to medium without addition of B73 stem material (red) (n = 2). (**B**) Scattered light (black) and Gfp fluorescence (green) monitoring of fungal growth in medium supplemented with 20 g/L of B73 (filled icons) or *bm3* (white icons) plant material as carbon source. The data are shown as the results of two independent fermentation experiments with independent fungal inoculums and 4 or 6 plants of B73 or *bm3*, respectively. The AVG ± SD is calculated from the resulting n = 8 and n = 12 for B73 and *bm3*, respectively. (**C**) pH (blue) and oxygen transfer rate (OTR; brown) monitoring of fungal growth in medium supplemented with 20 g/L of B73 (filled icons) or *bm3* (white icons) plant material as carbon source. The pH data are shown as the results of the same two independent fermentation experiments as scattered light and Gfp fluorescence, resulting in n = 8 and n = 12 for B73 and *bm3*, respectively. The OTR was monitored during one fermentation and the data are the AVG ± SD of n = 4 and n = 6 replicates for B73 and *bm3,* respectively

**Fig. 2:**
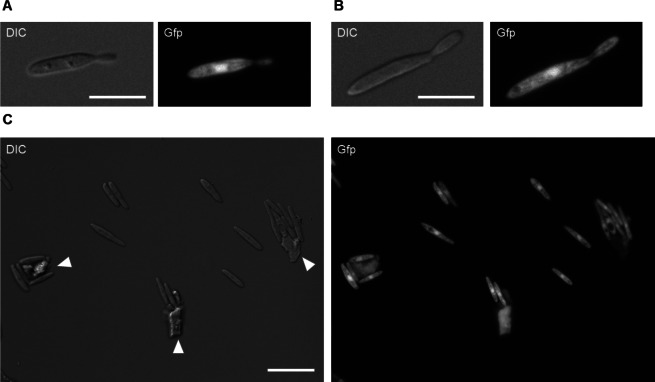
Growth of *U. maydis* ^Gfp^P_oma_bgl1 on corn stover. The used strain produced cytoplasmic Gfp accumulating in the cytosol and nucleus. (**A**) Cultivation of *U. maydis* with glucose as single carbon source visualized 16 h post inoculation (dividing cell). Scale bar, 10 µm. (**B**) Cultivation of *U. maydis* with corn stover as single carbon source visualized 16 h post inoculation (dividing cell). Scale bar, 10 µm. (**C**) Cultivation of *U. maydis* with corn stover as single carbon source visualized 16 h post inoculation (culture overview). White arrowheads indicate autofluorescent biomass particles. Scale bar, 20 µm

The ability of the fungus *U. maydis* to grow on increasing amounts of dried, milled corn stalks was investigated (Fig. 1A). The performance of *U. maydis* was assessed online using a BioLector® system, where scattered light was quasi-continuously monitored to measure the media's turbidity. This measurement served as a proxy for *U. maydis* growth as scattered light correlates with cell density (Samorski et al. 2005). The previously characterized *U. maydis* P_oma_bgl1 strain (Geiser et al. 2016) was modified to express the green fluorescent protein (Gfp) as a second method to monitor and quantify fungal growth. In the control with fungal inoculum but without corn stover, only a slight uptick in the scattered light was observed, reaching a maximum of 13.9 ± 0.1 a.u. after 22 h. This increase is likely due to residual nutrients still present in the inoculum media. In contrast, when the media was supplemented with corn stover, there was a notable increase in the scattered light, indicative of fungal biomass production. This surge began around 6 h after inoculation in all corn stover concentrations tested. While the maximum scattered light values were observed after 23 h using 3 g/L or 10 g/L corn stover, increasing the corn stover material to 20 g/L resulted in a maximum signal after 14 h (Fig. 1A). Microscopic inspection of the cultures after 16 h confirmed the presence of dividing fungal cells in the yeast form, mirroring cells grown in glucose (Glc) as sole carbon source (Fig. 2).

*U. maydis* growth performance on 20 g/L corn stover was further characterized by measuring metabolic activity parameters such as pH and oxygen transfer rate (OTR) in parallel to scattered light (Fig. 1B and C) combining BioLector® with µRAMOS technologies (Ladner et al. 2016). Measurements of the scattered light in cultures can be influenced by cell shape, cell size, and the corn stover particles in the system (Kunze et al. 2014). However, in the conditions tested, online monitoring of the Gfp fluorescence emitted by the employed *U. maydis* strain mirrored the scattered light curve. These results indicate that both methods can be used to estimate fungal growth.

Together with the initiation of the exponential growth phase after 6 h, the pH of the media decreased from its initial pH of 5.8 to a minimum of 5.4 after 12 h. This initial drop in pH may be indicative of increased metabolic activity involving for example the production of organic acids, or the release of acetic acid resulting from the breakdown of plant wall material. Aligning with the onset of the stationary growth phase, the pH of the media began to rise, and reached a 6.3 value by the end of the cultivation period (Fig. 1C). A similar shift in pH in *U. maydis* fermentations have been interpreted as a fungal response to nutrient limitations characterized by a transition to less acidic metabolic pathways (Geiser et al. 2014; Terfrüchte et al. 2018).

The OTR rapidly increased during the exponential growth phase consistent with an increase in metabolic activity of the fungus, as *U. maydis* metabolizes substrates and actively replicates, consuming oxygen. The OTR reached its maximum after 14 h, coinciding with the maximum fungal cell density determined by Gfp fluorescence and scattered light measurements. After a short plateau, the OTR decreased rapidly suggesting a decline in the metabolic activity (Flitsch et al. 2016; Ladner et al. 2016). OTR values reached a basal level after 20 h, which remained constant until the end of cultivation (Fig. 1C). The Gfp fluorescence signal increased correlating with the increment in scattered light signal, further confirming fungal proliferation and its quantitative traceability in the developed system (Fig. 1B). Collectively, these results show that *U. maydis* can utilize corn stover as a carbon source. Moreover, the developed screening method allows a detailed characterization of the fungal performance on corn stover in a microtiter scale by simultaneously recording diverse growth (scattered light and Gfp) and metabolic activity (OTR and pH) parameters online.

## *U. maydis* exhibits differential utilization of the various carbohydrate substrates present in corn stover

To identify the corn stover components utilized by *U. maydis*, we conducted a comprehensive compositional analysis of the residue remaining after incubation in the presence or absence of *U. maydis*. First, the suspensions were collected from the BioLector® plate at the end of the cultivation and the liquor was separated from the solid and dried before performing the analyses. The unfermented liquor fraction contained large amounts of soluble sugars, mainly glucose, sucrose and fructose (Table 1). However, after fermentation, only traces of these carbohydrates were detected, suggesting that *U. maydis* is able to utilize them as a carbon source. As corn stover is often autoclaved to avoid microbial contaminations during fermentation, we explored if this process alters the composition and availability of soluble sugars, but no significant differences were detected in non-autoclaved corn stover (Table 1).

**Table 1:** Carbohydrate composition of the liquor fraction [% of dry weight]

Condition	Glucose	Sucrose	Fructose	Total
B73 not autoclaved	9.1 ± 1.1	2.3 ± 1.1	9.3 ± 1.0	20.7 ± 1.4
B73—*U. maydis*	9.0 ± 0.9	2.4 ± 0.9	9.6 ± 0.6	21.0 ± 0.8
B73 + *U. maydis*	**0.3 ± 0.04**	**0.1 ± 0.03**	**0.04 ± 0.03**	**0.4 ± 0.05**
*bm3* not autoclaved	14.1 ± 0.8	3.6 ± 0.9	12.9 ± 1.0	30.5 ± 1.6
*bm3*—*U. maydis*	14.6 ± 0.5	3.0 ± 0.8	13.7 ± 0.8	31.2 ± 1.4
*bm3* + *U. maydis*	**0.3 ± 0.1**	**0.03 ± 0.01**	**0.2 ± 0.1**	**0.5 ± 0.1**

Soluble sugar quantification [% of dry weight] of not autoclaved, unfermented (- *U. maydis*) and fermented (+ *U. maydis)* B73 and *bm3* material. Data of the not autoclaved and unfermented soluble sugar contents are shown as AVG ± SD of n = 4 (B73) and n = 6 (*bm3*) plants. The data for the fermented soluble sugar contents are the results of two independent fermentation experiments with independent fungal inoculums and 4 or 6 plants of B73 or *bm3*, respectively. The AVG ± SD is calculated from the resulting n = 8 and n = 12 for B73 and *bm3*, respectively. Bold values indicate statistically significant differences between -/ + *U. maydis* conditions determined by pairwise comparisons of not autoclaved vs. unfermented and unfermented vs. fermented biomass by a two-tailed students t-Test at *p*-value < 0.05

The compositional analysis of the solid residue needs to consider the accumulation of fungal biomass as a result of the extensive generation of *U. maydis* biomass during fermentation. Quantification of glucosamine (GlcN), a monosaccharide present in *U. maydis* chitinaceous cell walls but absent in plant tissues, was used to monitor the amount of *U. maydis*-derived material in the residue. Monosaccharide composition of fungal biomass produced in media containing glucose as carbon source indicated the presence of GlcN, Gal and Glc. After subtraction of the *U. maydis* derived Glc and Gal, the relative abundance of plant-derived hemicellulosic monosaccharides within the solid residue was determined. This fraction was also analyzed for the presence of the main insoluble corn stover constituents *i.e.*, starch, lignin and crystalline cellulose (Table 2).

**Table 2:** Relative biomass composition [% of AIR] of the pre- and post-fermentation residue

Condition	Arabinose	Galactose	Glucose	Xylose	HC	CC	Lignin	Acetate	Starch	*U. maydis*	Total
B73—*U. maydis*	2.5 ± 0.1	0.7 ± 0.0	2.5 ± 0.4	23.1 ± 0.7	28.9 ± 0.7	35.1 ± 0.6	15.8 ± 0.9	5.0 ± 0.3	0.5 ± 0.2	n.d	85.3 ± 1.7
B73 + *U. maydis*	2.5 ± 0.1	**1.0 ± 0.1**	2.8 ± 0.3	**18.3 ± 0.7**	**24.6 ± 0.6**	**32.6 ± 1.2**	16.4 ± 0.2	**3.9 ± 0.2**	0.8 ± 0.0	11.2 ± 1.4	89.5 ± 1.2
bm3—*U. maydis*	2.4 ± 0.2	0.6 ± 0.0	2.1 ± 0.3	23.6 ± 0.8	28.7 ± 0.8	35.4 ± 1.5	15.4 ± 1.2	5.0 ± 0.2	0.4 ± 0.2	n.d	84.9 ± 2.9
bm3 + *U. maydis*	2.2 ± 0.1	**1.0 ± 0.1**	**3.0 ± 0.2**	**18.0 ± 1.0**	**24.2 ± 1.1**	**33.3 ± 0.8**	14.2 ± 0.8	**3.5 ± 0.2**	**0.7 ± 0.0**	15.4 ± 1.5	94.6 ± 1.7
B73—Celluclast®	1.7 ± 0.2	0.9 ± 0.1	2.5 ± 0.6	18.2 ± 1.6	23.2 ± 2.2	32.2 ± 2.7	15.3 ± 0.7	4.4 ± 0.3	0.7 ± 0.0	12.6 ± 1.5	88.4 ± 1.8
B73 + Celluclast®	**1.2 ± 0.1**	**0.6 ± 0.1**	**0.3 ± 0.3**	**14.7 ± 1.0**	**16.9 ± 1.2**	**27.5 ± 1.8**	**16.6 ± 0.9**	4.5 ± 0.7	**0.8 ± 0.1**	**24.1 ± 1.9**	90.4 ± 2.1
bm3—Celluclast®	1.2 ± 0.1	0.8 ± 0.1	2.2 ± 0.4	15.9 ± 0.8	20.2 ± 1.0	31.7 ± 1.9	12.8 ± 0.8	4.4 ± 0.5	0.7 ± 0.1	18.2 ± 2.1	87.9 ± 2.2
bm3 + Celluclast®	**0.9 ± 0.1**	**0.5 ± 0.1**	n.d	**12.9 ± 1.1**	**13.6 ± 1.7**	**25.1 ± 2.3**	**15.3 ± 0.5**	4.6 ± 0.4	**0.9 ± 0.1**	**37.4 ± 5.7**	96.9 ± 5.1

n.d. = not detected; HC = sum of hemicellulosic monosaccharides; CC = crystalline cellulose; Total = Sum of plant and fungal components

Comparison before and after digestion with *Ustilago maydis* and without/with addition of Celluclast®. Data without Celluclast® are the results of two independent fermentation experiments with independent fungal inoculums and 4 or 6 plants of B73 or *bm3*, respectively. The AVG ± SD is calculated from the resulting n = 8 and n = 12 for B73 and *bm3*, respectively. The addition of Celluclast® is shown as AVG ± SD of two technical replicates for each individual plant, *i.e.* n = 8 and n = 12 replicates for B73 and *bm3*, respectively. Bold values indicate statistically significant differences between the material determined by a two-tailed students t-Test at *p*-value < 0.05

The results did not reveal any significant decrease in the proportion of lignin between fermented and non-fermented residues, indicating that *U. maydis* is not able to degrade this aromatic polymer. Likewise, it appears that *U. maydis* does not utilize starch, although it should be noted that only trace amounts of this polymer are found in senescent corn stem tissue. Only a modest decrease in the relative abundance of crystalline cellulose (− 7.1 %) and total hemicellulose (− 14.9 %) was detected in fermented samples. Further determination of the hemicellulosic monosaccharide composition revealed that the reduction in total hemicellulose was likely caused by a decrease in xylose (− 20.8 %), indicating partial xylan degradation. Additionally, the strongest decrease was observed in the proportion of wall-bound acetate (− 22 %), which is mostly found as a substituent on the xylan backbone in corn stover (Table 2). The employed method thus provides detailed compositional residue quantification and reliably estimates the fungal abundance within the suspension (Table 2; Additional file 1: Table S1). The utilized method can only determine relative abundances, as the quantitative harvest of the solid fraction from the BioLector® plate is impossible. Hence, to validate our relative estimations, parallel cultures in glass shake flasks, compatible with mass balance calculations, were used. The analysis of the residue confirmed that the largest remaining fraction of the corn stover consisted of solid material. In fact, the percentage of solid residue increased from 67.8 ± 2.7 wt% in samples without *U. maydis* to 80.1 ± 1.2 wt% in fermented samples containing *U. maydis*. This considerable difference can be explained by the conversion of virtually all soluble sugars present in the liquor fraction into insoluble fungal materials. Further dissection of the solid fraction showed that the predominant portion in both conditions corresponded to alcohol insoluble residue (AIR), which mainly encompasses polysaccharides and other large polymers contained in plant and microbial cell walls. 11.2 ± 0.1 % of the AIR corresponded to *U. maydis* biomass, which matches the fungal biomass estimated in the BioLector® samples (11.2 ± 1.4 %) (Table 2, Additional file 2: Figure S2).

This analysis further confirmed the main results observed in the microtiter plates. The amount of crystalline cellulose in fermented samples decreased by 11.1 %. Although the reduction in total hemicellulose was not statistically significant, xylose and acetate content decreased by 15.8 % and 22.3 %, respectively, further suggesting degradation of O-acetylated xylan. Interestingly, neither of the two applied methods could account for the small but significant increase in galactose. One interpretation is that this increase is derived from the fungal cell wall, suggesting potential changes in the fungal wall composition if growing on corn stover compared to our reference glucose substrate.

Overall, these results indicate that *U. maydis* is mainly feeding on easily accessible soluble carbohydrates present in the plant biomass explaining the quick growth and metabolic activity rise during the initial stages of fermentation. Utilization of larger, insoluble lignocellulosic polymers seems to be limited. Lignin content remains unaffected and only a small fraction of crystalline cellulose and *O*-acetylated GAX appears to be susceptible to degradation and further utilization by the fungus under the used conditions, which aligns to the described hydrolytic enzyme repertoire secreted by *U. maydis*. This restricted availability of carbohydrate substrates from the lignocellulosic composite would explain the growth arrest and the drop in the fungal metabolic activity in the late fermentation stages observed by online monitoring.

## Growth performance of *U. maydis* is enhanced on corn stover derived from a maize lignin mutant

We proceeded to assess whether alterations in the lignocellulose structural attributes of the corn stover feedstock could enhance the utilization of this residue by *U. maydis*. Our initial investigation focused on the impact of a plant wall material with altered composition. For this purpose, we examined *U. maydis* performance growing on corn stover derived from a lignin-deficient maize mutant, since modification of the lignin composition has been shown to impact the enzymatic degradation of corn stover (Christensen and Rasmussen 2019). The *brown midrib 3* (*bm3*) maize mutant exhibits an increased ratio of guaiacyl to syringyl moieties in its lignin, resulting in enhanced enzymatic degradation (Santoro et al. 2010; Wang et al. 2023). When *bm3* corn stover was used as a substrate for *U. maydis* growth in our screening platform, a significant increase in the maximum scattered light signal was observed compared to the reference B73 corn stover previously used (Fig. 1B and C). Although there were no discernible differences in initial fungal growth between B73 and *bm3* material, the exponential phase was prolonged by 2 h on *bm3* material and the scattered light reached a maximum value 17.4 % higher than in B73. These findings were further corroborated by an increase in maximum Gfp fluorescence when *bm3* corn stover was used. Similarly, the maximum OTR was higher for *bm3* corn stover material than for B73. During the first 12 h, the OTR curves are indistinguishable. After that period, while B73 material reached a plateau, the OTR for *bm3* continued to rise, peaking 14 h after inoculation. Subsequently, the OTR decreased rapidly, ultimately reaching a final plateau after 19 h, similar to the pattern observed for B73. Notably, the pH profile also differed between the two different substrates. During the initial incubation period, the pH levels decreased similarly in both conditions. However, the pronounced pH increase observed for B73 material during the exponential growth phase of *U. maydis* was delayed and of a lesser magnitude in the case of growth on *bm3* material. Both OTR and pH measurements suggest a prolonged fungal metabolic activity when growing on *bm3* material. Similar results were obtained in four independent experiments, validating that *U. maydis* performance is enhanced in corn stover obtained from the lignin-deficient *bm3* mutant compared to B73.

The composition of the *bm3* residue after incubation was analyzed, following a similar approach as the one used for wildtype corn stover (B73). The undigested *bm3* liquor contained 10 % more soluble carbohydrates than B73. Consistent with the observations for B73 material, only traces of glucose, sucrose and fructose were detected in the samples after incubation with *U. maydis*, further confirming that the fungus efficiently converts these soluble carbohydrates (Table 1).

After incubation with *U. maydis*, the *bm3* solid fraction contained 15.3 ± 1.5 % of fungal material, representing a 37.8 % increase compared to cultivations on B73 (Table 2) confirming that *U. maydis* can grow more efficiently on *bm3* corn stover. Compositional analyses of the residue revealed a reduction of 6 % in the crystalline cellulose relative content due to fermentation, slightly reduced compared to the 7.1 % shown by B73. Notably, utilization of GAX in *bm3* corn stover seems to be improved as higher reductions in total hemicellulose, xylose, and acetate content compared to B73 were observed (15.7 %, 23.7 %, and 30 %, compared to 14.9 %, 20.8 %, and 22 %, respectively).

Both the online measurements and the analytical quantification of fungal biomass present in the residue after fermentation substantiate the superior growth of *U. maydis* in *bm3* material compared to B73 (Fig. 1B and C; Table 2). A higher abundance of soluble sugars in *bm3* corn stover appears to account for most of this enhanced fungal performance. However, an improved utilization of *O-*acetylated xylan may also contribute to this phenomenon suggesting that the changes in lignocellulosic properties of this maize variety might be beneficial for fungal performance.

## Performance of engineered lignocellulolytic *U. maydis* strains growing on corn stover

Expression of most genes encoding lignocellulose hydrolytic enzymes in *U. maydis* remains low during non-pathogenic yeast-like growth, becoming active primarily during the plant infection phase (Doehlemann et al. 2008; Geiser et al. 2016). A collection of engineered *U. maydis* strains secreting intrinsic enzymes with diverse hydrolytic activities during non-pathogenic growth was tested in our conditions (Geiser et al. 2016). The P_oma_bgl1 strain produces an exo-β1-4-glucanase, enabling it to grow on cellobiose as a sole source of carbon and to release small amounts of glucose from microcrystalline cellulose (Geiser et al. 2016). Supernatants of another strain, P_oma_egl1, harbor endo-β1-4-glucanase activity on carboxymethylcellulose and regenerated amorphous cellulose (Schauwecker et al. 1995; Geiser et al. 2016). The third selected strain, P_oma_xyn11A, shows enhanced xylanase activity in culture supernatants, generating smaller oligomers from birch-wood xylan compared to the control (Geiser et al. 2013, 2016).

**Fig. 3:**
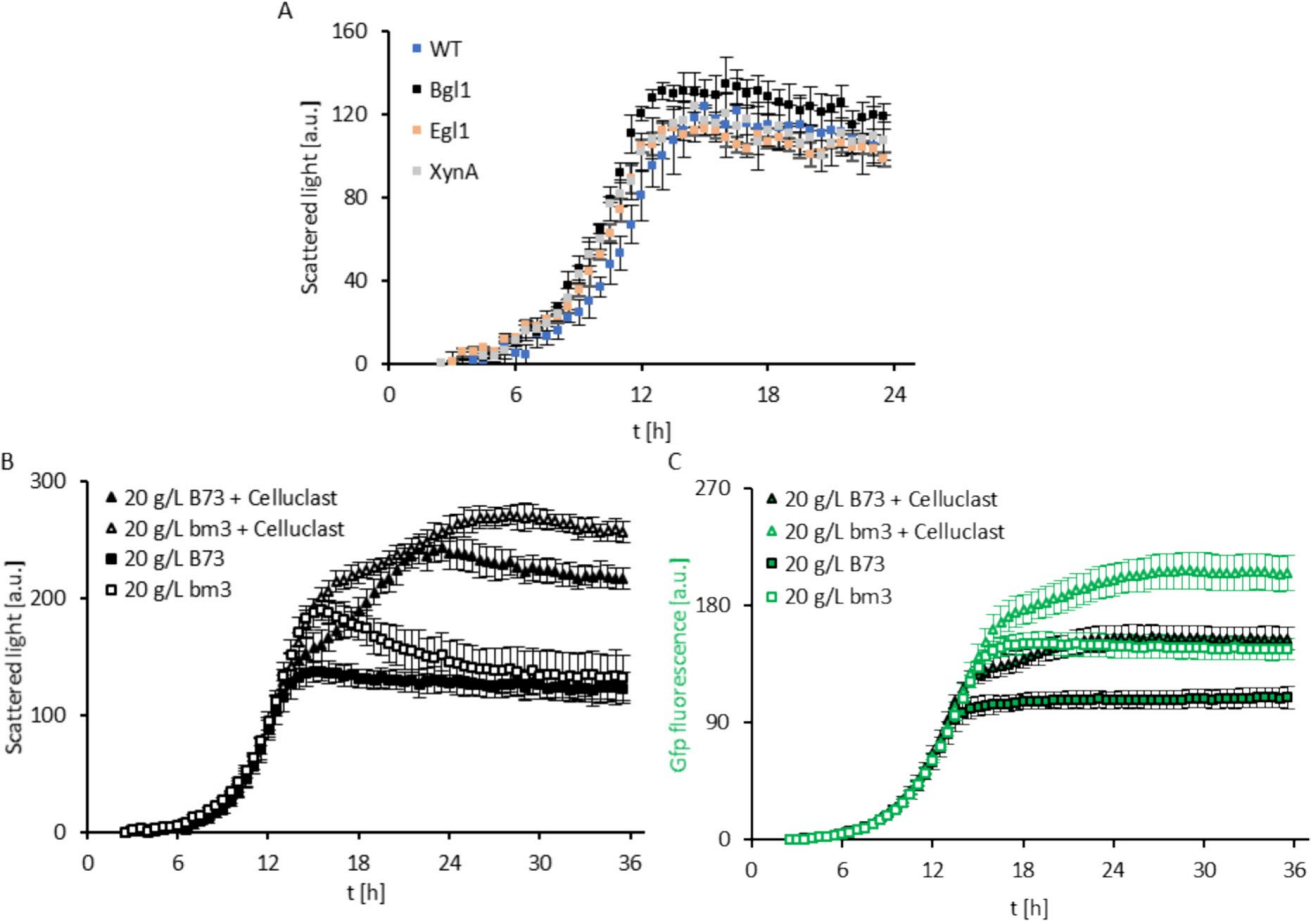
Online monitoring of biotechnological process optimization. (**A**) Scattered light monitoring of engineered *Ustilago maydis* MB215 derivatives growing on medium supplemented with 20 g/L B73 corn stover (n = 3 technical replicates)*.* Comparison of ^Gfp^P_oma_bgl1 (black), P_oma_egl1 (beige) and P_oma_xyn11A (grey). MB215^Gfp^ (WT, blue) was used as control. (**B**) Scattered light monitoring of fungal growth in medium supplemented with 20 g/L of B73 (filled icons) or *bm3* (white icons) corn stover as carbon source without (squares) and with (triangles) addition of Celluclast®. Data are shown as AVG ± SD of two technical replicates for each individual plant, *i.e.* n = 8 and n = 12 replicates for B73 and *bm3*, respectively. (**C**) Gfp fluorescence monitoring of fungal growth in medium supplemented with 20 g/L of B73 (filled icons) or *bm3* (white icons) corn stover as carbon source without (squares) and with (triangles) addition of Celluclast®. Data are shown as AVG ± SD of two technical replicates for each individual plant, *i.e.* n = 8 and n = 12 replicates for B73 and *bm3*, respectively

**Fig. 4:**
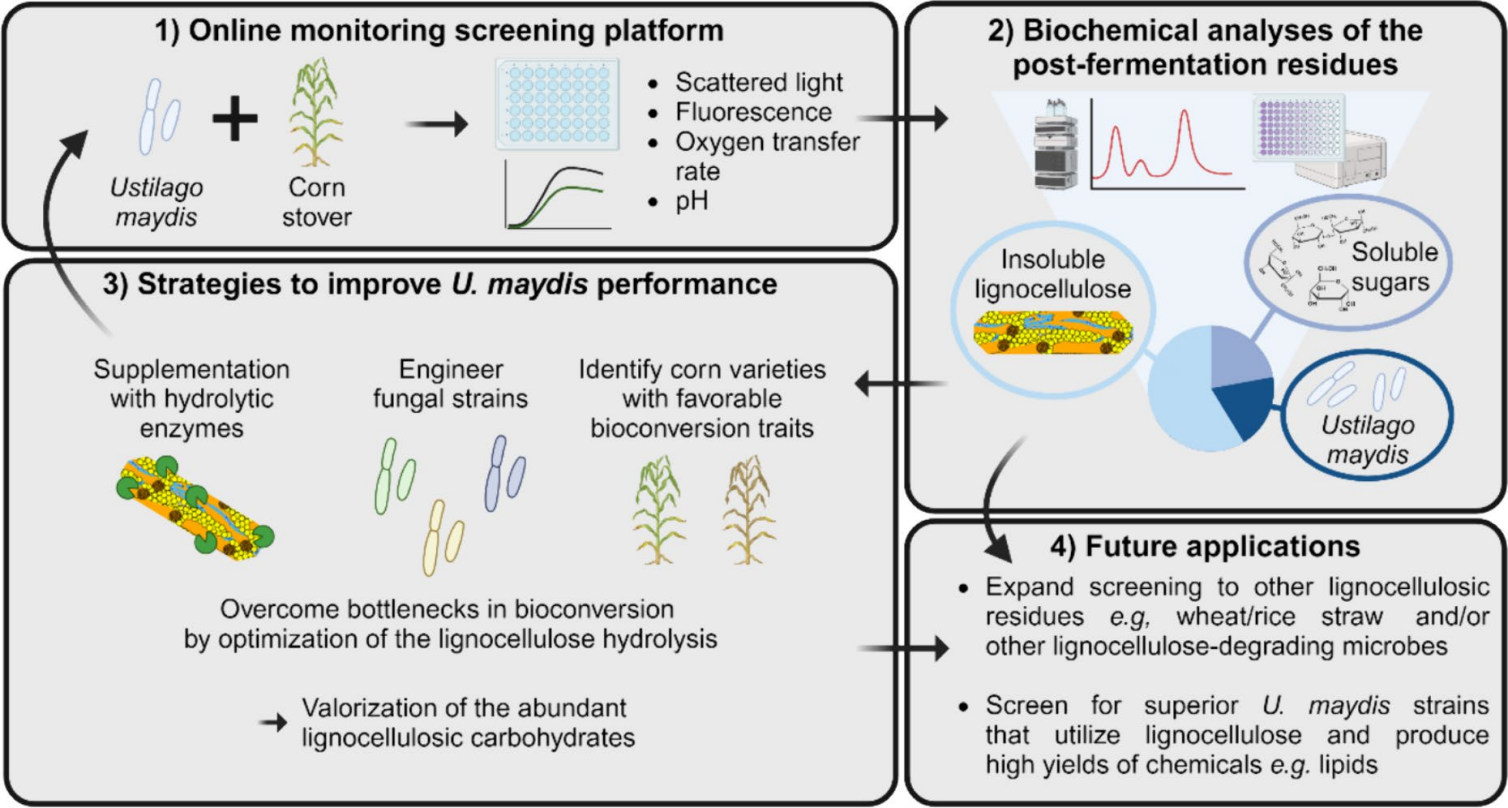
Schematic representation and prospective applications of the screening platform. (1) Simplified representation of the microtiter plate screening platform monitoring fungal growth (scattered light, Gfp fluorescence) and metabolic parameters (oxygen transfer rate, pH) while growing on corn stover. (2) Representation of the analytical pipeline identifying the specific carbohydrate sources utilized by *U. maydis*. (3) Three example approaches explored in this work to enhance the fungal performance on corn stover. 4) Prospective future applications of the developed screening platform

We assessed the performance of the three ^Gfp^P_oma_bgl1, P_oma_egl1 and P_oma_xyn11A *U. maydis* strains in our BioLector® screening platform comparing them to the control MB215^Gfp^ strain (Fig. 3A). All four strains showed similar overall performance when growing on corn stover. Growth acceleration was observed in each of the engineered strains, characterized by a shorter lag phase and faster reach of the maximum scattered light value compared to the control strain (Fig. 3A). The control strain entered the exponential growth phase around 6.5 h and reached maximum growth after 15.5 h. Meanwhile, P_oma_xyn11A, P_oma_egl1 and ^Gfp^P_oma_bgl1 strains initiated the exponential phase around 5 h and reached their respective maxima after 14.5 h, 13.5 h and 13 h. Notably, the ^Gfp^P_oma_bgl1 strain not only exhibited the fastest growth, but also achieved higher cell densities according to the scattered light measurements, indicating the best overall growth performance. The use of these strains did not show a clear improvement in lignocellulose utilization despite described enhanced lytic activities on artificial substrates. This suggests that increasing one single hydrolytic activity is not sufficient to enhance the digestion of the complex substrate corn stover. However, the results show the potential of the established BioLector®-based screening platform to assist in the rapid online evaluation of engineered *U. maydis* strains. Future work is needed to develop superior strains with an expanded repertoire of lignocellulolytic activities. The presented platform allows a parallel performance analysis of engineered *U. maydis* strains or consortiums expressing native and/or heterologous lignocellulolytic enzymes. Analysis of the post-fermentation residues, as described in this study, could be used to identify bottlenecks in the bioconversion process *e.g.*, degradation of specific polysaccharides into utilizable sugars (Fig. 4).

## Assisted enzymatic lignocellulose degradation improves *U. maydis* performance

After observing the limited utilization of the lignocellulosic fraction in our system, we tested whether the addition of an exogeneous enzyme cocktail with lignocellulolytic activity improves *U. maydis* performance. We evaluated the growth of *U. maydis* on corn stover (variety B73) comparing cultures with and without the addition of Celluclast® (Fig. 3B and C). Scattered light and Gfp fluorescence were monitored for up to 36 h allowing the detection of the plateau under these conditions. The initial exponential growth phase was identical independent of the addition of Celluclast®, reaching the stationary phase at 137 ± 9 a.u. after 15 h. However, the addition of Celluclast® resulted in a notable shift in fungal growth dynamics, characterized by a second, less rapid growth phase that led to a substantial increase in the maximum scattered light, reaching 242 ± 12 a.u. after 22 h. This corresponds to a 176.6 % increase in the fungal cell density directly attributable to the addition of Celluclast®. Measurement of Gfp fluorescence showed comparable results, and Celluclast®-supplemented samples exhibited a secondary boost in signal intensity mirroring the scattered light profile (Fig. 3C).

The analysis of the solid residues confirmed the online data, as the amount of fungal material present in the solid residue from samples with Celluclast® reached 24.1 %, almost doubling the amount observed in the non-supplemented control (Table 2). Celluclast® addition significantly decreased the relative abundances of all cell wall carbohydrate sources present in corn stover. The proportions of crystalline cellulose and total hemicellulose decreased by 14.6 and 27.1 %, respectively. All hemicellulosic monosaccharides were reduced, particularly glucose where only trace amounts were found in samples supplemented with Celluclast®.

Using *bm3* corn stover as substrate combined with Celluclast® addition resulted in synergistic effects (Fig. 3B and C). A first peak in maximum fungal growth was reached after 15.5 h, exhibiting higher scattered light and Gfp fluorescence compared to B73 as expected from better fungal performance. Celluclast® addition to *bm3* corn stover also resulted in a second, less rapid growth phase, but a higher maximum value was obtained and at a later time (27 h) compared to B73. Similar distinctions were identified when analyzing the Gfp fluorescence (Fig. 3C). Confirming the online measurements, the quantity of fungal biomass detected in *bm3* solid residue supplemented with Celluclast® reached 37.4 % representing a 105.5 % increase compared to non-supplemented *bm3* (Table 2). This increase in fungal biomass production resulting from Celluclast® addition was higher in *bm3* compared to B73. Similarly, the solid residue composition showed bigger reductions in the proportion of crystalline cellulose and hemicellulose components (Table 2). This suggests that the altered wall structure of *bm3* mediates enhanced substrate accessibility and/or hydrolytic activity of the enzymes present in the Celluclast® cocktail.

Unexpectedly, we also observed increments in the relative abundance of lignin and starch correlating with an increasing abundance of fungal biomass. One possibility is that these values reflect the relative decrease of the other components of the solid residue. Alternatively, we cannot discard the possibility that certain fungal components interfere with our determinations, primarily when large amounts of fungal biomass are present in the solid residue as in the case of *bm3* samples supplemented with Celluclast®. Fungal cell membranes contain sterols which may interfere with our spectrophotometric quantification of lignin (Baloch et al. 1984). Similarly, *U.* *maydis* might accumulate glycogen during corn stover fermentation which is indiscernible from starch in our assays.

Together, these results indicate that Celluclast® treatment during cultivation results in additional substrates derived from the lignocellulose fraction in corn stover for *U. maydis* to continue growing upon consumption of the soluble sugars. Combining the lignocellulolytic activity supplementation with the use of *bm3* corn stover results in a synergistic effect, allowing a threefold build-up of fungal biomass compared to the initial conditions set for B73.

## Conclusions

Our study describes the implementation of a microtiter plate screening platform for the analysis of *U. maydis* growing on corn stover. The method combines the online monitoring of fungal growth and metabolic parameters with compositional analysis of the pre- and post-fermentation residue, allowing for a detailed characterization of the microbe performance and the utilization of the diverse carbohydrate sources present. Our results demonstrate that *U. maydis* is able to utilize corn stover as the sole nutrient source. The quasi-continuous monitoring of scattered light, Gfp fluorescence, pH and OTR, along with the microscopic and biochemical estimation of the amount of fungal material in the post-fermentation residue allowed us to reach an unprecedented level of detail to profile fungal growth.

Additionally, the method allowed us to dissect the specific utilization of each of the diverse carbohydrate sources present in corn stover. Our data reveal that *U. maydis* mostly utilizes soluble sugars *i.e.,* glucose, sucrose and fructose when growing on corn stover, while only a small fraction of the lignocellulosic carbohydrates are hydrolyzed. This result might be unexpected, given the well documented repertoire of potential lignocellulose-degrading enzymes encoded in *U. maydis* genome (Mueller et al. 2008; Doehlemann et al. 2008; Couturier et al. 2012; Reyre et al. 2022). The low expression level of these lignocellulose-degrading enzymes under the fermentation conditions used might explain this observation. Although further research is necessary to explore this and other possibilities, these results highlight the biotechnological potential of enhancing degradation of the abundant lignocellulose materials contained in corn stover to improve fungal performance.

Even though the low overall substrate utilization ratio of corn stover by *U. maydis* in our system limits direct industrial applications, it also unveils prospective applications to improve the process by multiple angles. All approaches considered here were successful in boosting fungal performance but with varying degrees of effectiveness, with Celluclast® supplementation showing the best increase in fungal performance. In future applications, the method could also be used to study the effect of enzymes added exogenously to the fermentation reaction or secreted by *U. maydis* as the pipeline enables the investigation of enzyme properties including catalytic activity, substrate specificity or synergistic effects on complex natural lignocellulosic substrates.

The enhanced performance observed when using *bm3*-derived corn stover highlights that lignocellulose traits of the plant biomass affect the plant biomass bioconversion process. The semi-high throughput nature of the online monitoring method is compatible with large-scale screenings to evaluate the suitability of different plant biomass sources. The extensive diversity of genetic resources available for maize could be screened to identify favorable plant biomass bioconversion traits, such as a higher abundance of specific substrates, lower presence of inhibiting compounds, or modified wall architecture facilitating a better enzyme accessibility. Similarly, it could be applied to the assessment of mechanical, thermal, or chemical pretreatments aiming to change the structure, accessibility or degradability of the diverse lignocellulose components. In sum, the outlined screening method serves as the foundational framework for prospective applications of corn stover as a renewable substrate for consolidated bioprocessing involving *U. maydis* as a valuable tool to scale-up the fermentation from laboratory scale to production scales.

## Supplementary Information

Additional file 1

Additional file 2

## Data Availability

The datasets used and/or analyzed during the current study are available from the corresponding author on reasonable request.

## Abbreviations

µRAMOS: Micro respiratory activity monitoring system
AIR: Alcohol-insoluble residue
*bm3*: *brown-midrib 3*
CAZymes: Carbohydrate-active enzymes
CM: Complex medium
Gal: Galactose
GAX: Glucuronoarabinoxylan
Gfp: Green fluorescent protein
Glc: Glucose
GlcN: Glucosamine
HPAEC: High-performance anion-exchange chromatography
OD_600_: Optical density at 600 nm wavelength
OTR: Oxygen transfer rate
