# Supplementary material for: Monitoring corn stover processing by the fungus Ustilago maydis
Source: Bioresour Bioprocess. 2024 Sep 14;11(1):87. doi: 10.1186/s40643-024-00802-3 (PMC11401804; doi:10.1186/s40643-024-00802-3)
Supplement: Supplementary file 2 — Additional file 2 [file 40643_2024_802_MOESM2_ESM.docx]

**Supplementary materials**

**Monitoring corn stover processing by the fungus *Ustilago maydis***

Stefan Robertz^a, d^, Magnus Philipp^b, d, 1^, Kerstin Schipper^b, d^, Paul Richter^c, d^, Katharina Miebach^c, d^, Jorgen Magnus^c, d^, Markus Pauly^a, d^ and Vicente Ramírez^a, d, ^[[1]](#footnote-1)^^

^a^ Institute for Plant Cell Biology and Biotechnology, Cluster of Excellence on Plant Sciences, Heinrich Heine University Düsseldorf, 40225 Düsseldorf, Germany

^b^ Institute for Microbiology, Heinrich Heine University Düsseldorf, 40225 Düsseldorf, Germany

^c^ Aachener Verfahrenstechnik – Chair of Biochemical Engineering, RWTH Aachen University, 52074 Aachen, Germany

^d^ Bioeconomy Science Center (BioSC), Forschungszentrum Jülich, 53435 Jülich, Germany

Figure S1: Particle size distribution of the milled corn stover.

Milled and dried B73 corn stover resuspended in water was analyzed with a Leica DM2000 microscope and representative pictures taken with a Leica MC170HD camera. The sizes of 200 individual particles were measured with ImageJ (Version 1.54).

Figure S2: Glucosamine content in *Ustilago maydis* AIR and detailed mass balance of corn stover -/+ *Ustilago maydis.*


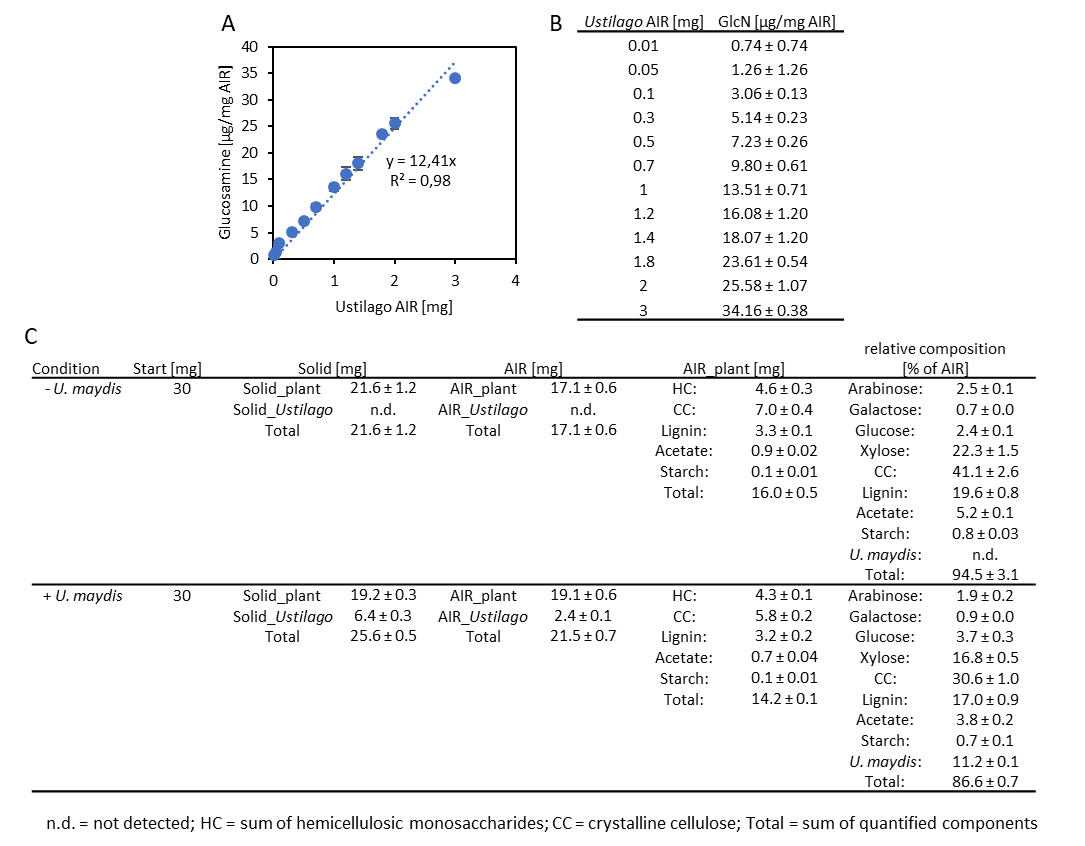


(A) Linear regression of glucosamine concentration detected per mg *Ustilago maydis* AIR. (B) Correlation of *Ustilago maydis* AIR to glucosamine concentration. Values are shown as mean ± SD of 3 *U. maydis* replicates. (C) Mass balance in glass flasks. Data are shown as mean ± SD of 3 technical plant replicates.

1. Corresponding author; Institute for Plant Cell Biology and Biotechnology, Heinrich Heine University, 40225 Düsseldorf, Germany; Email: ramirezg@hhu.de

   ^1^ Present address: Institute for Biotechnology and Foodscience, Norwegian University of Science and Technology, Gløshaugen, 7034 Trondheim, Norway [↑](#footnote-ref-1)
